# Genome-wide association mapping of resistance to *Phytophthora sojae* in a soybean [*Glycine max* (L.) Merr.] germplasm panel from maturity groups IV and V

**DOI:** 10.1371/journal.pone.0184613

**Published:** 2017-09-14

**Authors:** Jun Qin, Qijian Song, Ainong Shi, Song Li, Mengchen Zhang, Bo Zhang

**Affiliations:** 1 Department of Crop and Soil Environmental Sciences, Virginia Tech, Blacksburg, VA, United States of America; 2 National Soybean Improvement Center Shijiazhuang Sub-Center. North China Key Laboratory of Biology and Genetic Improvement of Soybean Ministry of Agriculture. Cereal & Oil Crop Institute, Hebei Academy of Agricultural and Forestry Sciences, Shijiazhuang, Hebei, P.R. China; 3 USDA, Agricultural Research Service, Soybean Genomics and Improvement Lab, Beltsville, MD, United States of America; 4 Department of Horticulture, University of Arkansas, Fayetteville, AR, United States of America; Agriculture and Agri-Food Canada, CANADA

## Abstract

*Phytophthora sojae*, an oomycete pathogen of soybean, causes stem and root rot, resulting in annual economic loss up to $2 billion worldwide. Varieties with *P*. *sojae* resistance are environmental friendly to effectively reduce disease damages. In order to improve the resistance of *P*. *sojae* and broaden the genetic diversity in Southern soybean cultivars and germplasm in the U.S., we established a *P*. *sojae* resistance gene pool that has high genetic diversity, and explored genomic regions underlying the host resistance to *P*. *sojae* races 1, 3, 7, 17 and 25. A soybean germplasm panel from maturity groups (MGs) IV and V including 189 accessions originated from 10 countries were used in this study. The panel had a high genetic diversity compared to the 6,749 accessions from MGs IV and V in USDA Soybean Germplasm Collection. Based on disease evaluation dataset of these accessions inoculated with *P*. *sojae* races 1, 3, 7, 17 and 25, which are publically available, five accessions in this panel were resistant to all races. Genome-wide association analysis identified a total of 32 significant SNPs, which were clustered in resistance-associated genomic regions, among those, ss715619920 was only 3kb away from the gene Glyma.14g087500, a subtilisin protease. Gene expression analysis showed that the gene was down-regulated more than 4 fold (log2 fold > 2.2) in response to *P*. *sojae* infection. The identified molecular markers and genomic regions that are associated with the disease resistance in this gene pool will greatly assist the U.S. Southern soybean breeders in developing elite varieties with broad genetic background and *P*. *sojae* resistance.

## Introduction

Phytophthora root rot, caused by a fungus *Phytophthora sojae* (*P*. *sojae*), is a major disease of soybean (*Glycine max*), especially in the areas where soybeans have been cultivated for many years. Yield loss can be substantial, and even entire fields may be destroyed [1]. Utilization of resistant cultivars is the most economical and environmental friendly method to control this disease [2]. It is confirmed that deployment of resistance to *P*. *sojae* genes (*Rps* genes) in soybean cultivars has been an effective method of controlling *P*. *sojae* [3].

*P*. *sojae* follows a gene-for-gene interaction and is hypothesized to have coevolved with its host plant soybean. At least 13 resistance *Rps* genes are known in soybean, and different races of *P*. *sojae* are able to overcome these resistance genes, both singly and in combination [4]. Linkage mapping and association mapping have been both conducted in *P*. *sojae* research to locate *Rps* genes and quantitative trait loci (QTLs). Bi-Parental mapping populations have been used to detect QTLs conferring partial resistance to *P*. *sojae*. For example, *Rps11* gene was mapped to a 225.3 kb genomic region on Chr 7 flanked by simple sequence repeat (SSR) markers using 58 F_2_ individuals and 209 F_2:3_ families derived from PI 594527, resistant gene donor, and the susceptible cultivar ‘Williams’ [5]. Two QTLs on Chr 19 were identified using a population of 246 RILs derived from Conrad × Sloan through sequence and gene expression analysis [6]. Association mapping, known as linkage disequilibrium (LD) mapping, is a powerful high-resolution mapping tool for complex traits within natural populations [7]. It detects and locates QTLs based on the strength of the correlation between mapped genetic markers and traits [8]. Four SSR markers on chr. 2 and 17 were identified to be associated with partial resistance to *P*. *sojae* race 2 using 175 soybean accessions by TASSEL (GLM) and (MLM) methods [9]. Fourteen single nucleotide polymorphism (SNPs) were also identified using Chinese soybean mini core collection of 224 accessions screened by 1,645 SNPs [10]. QTLs partially resistant to *P*. *sojae* were also located on Chrs 3, 13 and 19 through the study of 800 South Korean soybean accessions by linkage disequilibrium block analysis [11].

MGs IV and V soybeans are adapted to and widely grown in the Southern states such as Virginia, Arkansas and Tennessee. However, most *Rps* genes were identified in soybeans from MGs 000 to IV [12], and not many *P*. *sojae* resistant donors are evaluated for the adaptation to the South, which made it difficult to implement *P*. *sojae* resistant genes into the Southern soybean germplasm. Our study aimed to establish a *P*. *sojae* resistance gene pool that is high in genetic diversity to be employed in Southern soybean breeding programs, and to explore *P*. *sojae* resistance gene regions underlying the host resistance to *P*. *sojae* races 1, 3, 7, 17 and 25 for molecular breeding selection. The findings in this study will greatly help the Southern soybean breeders to develop elite varieties with broad genetic diversity and *P*. *sojae* resistance using identified SNPs markers specifically for this gene pool.

## Materials and methods

### Panel for genome-wide association analysis

All 6,749 MGs IV and V soybean accessions collected by National Genetic Resources Program were grouped into 500 clusters based on their genetic distance which were calculated using 42,509 SNPs that were included in the SoySNP50K [13, 14]. The accessions with the highest average genetic distance within each cluster were selected to form a MGs IV and V core collection consisting of 500 accessions. Among these, the panel of 189 accessions with public *P*. *sojae* data (http://www.ars-grin.gov) were chosen for this study. These accessions are original from 10 countries including China (140/189, 74.1%), Japan (17/189, 9.0%), South Korea (16/189, 8.8%), United States (6/189, 3.2%), and a few other countries (S1 Table).

### Disease assays and phenotypic data

The responses of 189 soybean accessions to races 1, 3, 7, 17 and 25 of *P*. *sojae* were evaluated by USDA-ARS germplasm curation program led by Dr. Randy Nelson and their collaborators at University of Kentucky, and University of Minnesota. The disease evaluation was conducted in greenhouses in various years. Briefly, young plants with the first unifoliate leaves just opened were inoculated using hypocotyl inoculation technique [4]. The ratings were recorded on each pot consisting of ten seeds of each accession at 4 to 5 days after inoculation. Pots containing greater than 70%, 30% -70% and less than 30% living plants or plants with non-killing lesions were rated as resistant (R), heterogeneous (H) and susceptible (S), respectively. Only 189 soybean accessions rated as resistant, and susceptible were chosen to conduct GWAS analysis (S2 Table). Association mapping for each race were conducted separately using “1” as R (resistant) and “9” as S (susceptible). Resistant and susceptible checks were included in each test to evaluate the virulence of the pathogen. Detailed protocol and references can be found in GRIN (https://npgsweb.ars-grin.gov/gringlobal/descriptors.aspx).

### Genotypic data

The 189 soybean accessions were genotyped with SoySNP50K beadchip, containing 52,041 SNPs [13]. Genotyping of the SNPs followed the procedure described by Song et al. (2013). The genotypic dataset contained 42,291 SNPs across 189 accessions after filtering SNPs with missing data and greater than 10% heterozygosity [14]. SNPs with minor allele frequencies lower than 5% were also discarded. The remaining 33,625 high quality SNPs were used for population structure and association analysis. Physical position of the SNPs was originally based on genome assembly version Glyma1.01 [14] and then was determined based on the Glyma.Wm82.a2.v1 [15].

### Population structure, genetic diversity and association analysis

STRUCTURE, a program that uses Bayesian method to analyze multi-loci data in population genetics [16], was used to analyze population structure and to create Q-matrix for association analysis. A hybrid model and an allelic variation occurrence, non-correlative model were used to examine the population structure of soybean germplasm. The number of the subpopulation (K) was assumed to be between 1 and 12. Thus, each K was run 10 times, the Markov Chain Monte Carlo (MCMC) length of the burn-in period was set to 20,000 and the number of MCMC iterations after the burn-in was also set to 20,000. For each simulated K, the statistical value delta K was calculated using the formula described by Evanno et al. [17]. The optimal K was determined using STRUCTURE HARVESTER [18] (http://taylor0.biology.ucla.edu/structureHarvester/). After the optimal K was determined, a Q-matrix was obtained and used in TASSEL 5 [19] for association analysis. Each soybean accession was then assigned to a cluster (Q) based on the probability that the genotype belonged in that cluster. The cut-off probability for assignment to a cluster was 0.5 for clusters (structure populations). Based on the optimum K, a Bar plot with ‘Sort by Q’ was obtained to visualize the population structure among the 189 accessions. Genetic diversity was also assessed and the phylogeny tree was drawn using MEGA 6 [20] based on the Maximum Likelihood (ML) tree method [21]. For sub-tree of each Q (cluster), the shape of ‘Node/Subtree Marker’ and the ‘Branch Line’ was drawn with the same color as in the figure of the Bar plot of the population clusters from the STRUCTURE analysis.

Association mapping of *P*. *sojae* resistance to each of the *P*. *sojae* races 1, 3, 7, 17, and 25 was conducted separately based on three different models in TASSEL 5: 1) the single marker regression (SMR) model without structure and without kinship, 2) the regression linear model (GLM-Q), and 3) the mixed linear model (MLM-Q+K) methods as described by Bradbury et al (2007) [19] (http://www.maizegenetics.net/tassel). Based on the suggestion by Lander and Botstein (1989) [22], a typical LOD threshold was usually between 2 and 3 in order to detect at 5% level for a QTL [23]. The SNPs were selected as the significantly associated with *P*. *sojae* resistance based on three models with the LOD threshold greater than 3.0 in this study to increase the likelihood of linkage.

### Gene expression analysis

Published gene expression data generated by RNA sequencing were downloaded from GEO database (GSE48524). Gene expression analysis was performed according to the method in the original publication [24]. Briefly, 10 isogenic lines were treated by *P*. *sojae* race 1, and differentially expressed genes were identified using edgeR with FDR < 0.05. These differentially expressed genes were compared to the significant SNPs found in the genome. For each significant SNP identified in this GWAS study and the significantly differentially expressed genes near the SNPs were identified based on the SNPs position in the whole genome sequences (Table 1).

**Table 1:** SNPs and significant regions associated with *P*. *sojae* races 1, 3, 7, 17 and 25 (P<0.001).

SNPs type	Chr.	SNP position (Glyma.Wm82.a2.v1)	SMR (LOD)	GLM (Q) (LOD)	MLM (Q+K) (LOD)	*P*. *Sojae* race	Significant regions	Gene Name	Distance to SNP(bp)	Annotation
A/G	3	3889598	5.12	5.82	3.57	1,7	sr3-race1&7	Glyma.03g034400	136,614	LRR and NB-ARC domains-containing disease resistance protein
C/T	5	41615242	3.25	4.56	4.20	1	sr5-race1	Glyma.05g209300	2,484,654	Disease resistance protein (TIR-NBS class)
A/C	5	41648449	3.16	4.42	3.87	1	sr5-race1	Glyma.05g213400	2,155,204	Disease resistance-responsive (dirigent-like protein) family protein
A/G	13	29036850	4.01	4.35	3.76	1	sr13-race1	Glyma.13g184800	821,150	LRR and NB-ARC domains-containing disease resistance protein
A/C	13	29042122	4.18	4.46	4.03	1	sr13-race1			
A/G	18	54969812	5.45	4.14	3.35	1	sr18-race1			
C/T	7	185603	4.40	4.01	3.43	3	sr7-race3	Glyma.07g007800	389,555	Disease resistance protein RPS4-RELATED
A/G	3	4482448	3.68	3.09	3.02	7	sr3-race7	Glyma.03g037000	33,508	LRR and NB-ARC domains-containing disease resistance protein
A/G	4	47077729	4.36	3.18	3.03	7	sr4-race7			
A/G	4	47313368	5.02	4.06	3.30	7	sr4-race7			
G/T	4	47321023	4.36	3.35	3.28	7	sr4-race7			
G/T	4	47329974	4.36	3.35	3.28	7	sr4-race7			
A/C	4	47344338	4.55	3.36	3.19	7	sr4-race7			
G/T	4	47346053	5.90	4.72	4.02	7	sr4-race7			
A/G	4	47350352	5.21	4.04	3.72	7	sr4-race7			
G/T	4	47367432	4.56	3.63	3.41	7	sr4-race7			
A/G	4	47371413	4.62	3.61	3.40	7	sr4-race7	Glyma.04g205200	412,544	Defense response
C/T	13	10487933	4.13	3.52	3.28	7	sr13-race7	Glyma.13g028100	3,158,795	Disease resistance protein RPS4-RELATED
C/T	13	9381699	4.39	3.31	3.40	7	sr13-race7			
A/G	3	31136061	3.67	4.41	3.70	17	sr3a-race17	Glyma.03g149600	5,355,720	Resistance to phytophthora 1
C/T	3	5486669	6.90	6.02	4.18	17	sr3b-race17			
C/T	10	39347629	5.88	5.17	3.24	25	sr10-race25	Glyma.10g127500	5,472,726	Disease resistance-responsive (dirigent-like protein) family protein
A/G	10	39354992	5.80	5.13	3.25	25	sr10-race25			
A/G	10	39358965	5.88	5.17	3.24	25	sr10-race25			
C/T	10	39364212	5.88	5.17	3.24	25	sr10-race25	Glyma.10g129400	4,701,960	Disease resistance family protein / LRR family protein
A/G	10	39365693	5.88	5.17	3.24	25	sr10-race25	Glyma.10g184300	2,381,651	Disease resistance protein RPS4-RELATED
C/T	10	39376495	5.88	5.17	3.24	25	sr10-race25			
C/T	10	39394332	5.88	5.17	3.24	25	sr10-race25	Glyma.10g196700	3,413,204	Disease resistance protein (CC-NBS-LRR class) family
A/G	14	7839347	6.73	6.45	5.55	25	sr14-race25	Glyma.14g079500	1,005,837	Arabidopsis broad-spectrum mildew resistance protein RPW8
G/T	14	7853431	6.73	6.43	5.50	25	sr14-race25	Glyma.14g079600	1,012,545	Arabidopsis broad-spectrum mildew resistance protein RPW8
A/G	14	7861677	7.97	7.49	6.05	25	sr14-race25			
A/G	18	53821341	5.62	5.03	3.18	25	sr18-race25			

## Results and discussion

### Phenotypic variation

A total of 189 accessions from the soybean panel of MGs IV and V were analyzed for the resistance to *P*. *sojae* races 1, 3, 7, 17 and 25 (S1 Table). Among the 189 accessions, all of them had phenotypic data of responses to *P*. *sojae* race 1, 119 to race 3, 89 to race 7, 81 to race 17, and 85 to race 25. The ratio of resistant accessions to susceptible accessions was 81/108 (0.75), 48/71 (0.68), 27/63 (0.43), 25/56 (0.45), and 21/65 (0.32) for the resistance to *P*. *sojae* races 1, 3, 7, 17, and 25, respectively, indicating there were relatively more accessions that are resistant to *P*. *sojae* race 1, and fewer accessions that are resistant to race 25. Five out of 189 accessions showed resistance to all five *P*. *sojae* races, and among the five accessions, PI 567780B, PI 567781, PI 587612A, and PI 588021A were collected from China, and PI 378682C from Japan (S2 Table). We also found ten accessions resistant to four *P*. *Sojae* races, 11 resistant to three races, 32 resistant to two races, and 39 resistant to only one race (S2 Table). Those accessions, particularly the five accessions resistant to all races, can be potential parental lines to use by the Southern soybean breeding programs to develop *P*. *sojae* resistant varieties.

The most resistant accessions in this panel were mainly from China. Among the 97 resistant soybean accessions, 78 accessions are from China and seven from Japan, nine from South Korea, two from the U.S., and one is unknown. Huang et al. (2016) also observed a total of 168 (75%) soybean accessions showed resistance to more than one *P*. *sojae* race and suggested that abundant resistant resources existed [10]. A systematic and effective evaluation 500 accessions for *P*. *sojae* resistance may result in new novel resistant genes.

### Population structure

**Fig 1:**
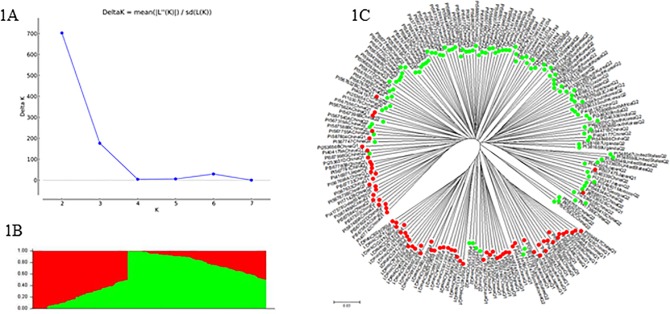
Model-based population structure for the soybean panel: (A) Delta K values for different numbers of populations assumed (K) in the STRUCTURE analysis, (B) phylogenetic tree constructed by neighbor-joining (NJ) of genetic distance by MEGA 6, and (C) Classification of two populations using STRUCTURE 2.3.4. The distribution of the accessions to different populations is indicated by the color code (Q1: red and Q2: green), consistent in the figures (B) and (C).

The 189 accessions were divided into two sub-populations (clusters), Q1 (red) and Q2 (green) based on STRUCTURE analysis as the maximal delta K value was observed when K = 2 (Fig 1A and 1B). They were also clustered into two major clusters. Result showed that the individuals in each of the two sub-populations from Structure analysis were consistent with that in each of the two groups from cluster analysis. Cluster I was comprised of 77 accessions, among which 67 accessions were collected from China, seven from Japan, two from the South Korea and one from U.S. Cluster II included 112 accessions including 76 from China, 13 from South Korea, nine from Japan, six from the US, two from India, one from each of the four countries, Morocco, Nepal, South Africa, and Vietnam. The accessions from China were the majority in this study, 75.7% of the total accessions, 87.0% in cluster I and 67.9% in cluster II. In addition, the accessions from other countries were also distributed in two clusters (Fig 1C, S1 Table). The correlation coefficient of 0.227 between clusters (Q1/Q2) and MGs (IV/V) (P = 0.0017) indicated that maturity group may affect the clustering of all accessions. Cluster Q1 (77) was composed of 72.72% MGs IV (56/77 = 72.72%) and 27.28% MGs V, and cluster Q2 (112) was composed of 50% MGs IV (56/112 = 50%) and 50% MGs V.

### Association analysis and SNP markers identification

A total of 32 SNPs (p< = 0.001, LOD> = 3) were identified to be associated with the resistance to at least one of the five tested *P*. *sojae* races 1, 3, 7, 17, and 25 (Table 1). They were grouped into thirteen significant regions covering the genome size from 1.5 to 46 kb (Table 1). The significant region in this research was named as combined chromosome number and race name, for example, ss715592224 is named as sr15-race1.

We identified five SNPs associated with race 1, one SNP associated with race 3, twelve SNPs associated with race 7, two SNPs associated with race 17, eleven SNPs associated with race 25 and one SNP associated with both races 1 and 7 The highest LOD values among all races ranged from 3.42 to 6.05 in MLM analysis (Table 1). For example, ss 715619926, associated with race 25, had the highest LOD of 6.05 in MLM analysis, and it had the highest LOD of 7.97 and 7.49 in SMR and GLM analysis, respectively. The highest LOD values from SMR and GLM are generally higher than those from MLM as MLM provides more stringent control over the population structure. Because some of the significantly associated SNPs were close to each other, genomics regions of these SNPs were delimited such that they were grouped in regions with significant associations. For example, s715592224 and ss715592229 on Chr. 5 were grouped in a 33 kb significant region named after sr5-race1. ss715614941 and ss715614943 on Chr. 13 were grouped in a 5 kb significant region named after sr13-race1. Therefore, race 1 had three, race 3 had one, race 7 had three, race 17 had two, and race 25 had three significant regions, and races 1 and 7 shared one significant region. (Table 1).

Six SNPs were associated with race 1 and they were located on Chr. 3, 5, 13, and 18 (Table 1). Ss715614943 on Chr.13 had the highest significant association with *P*. *sojae* race 1 with a LOD value of 4.18 in SMR, 4.46 in GLM, and 4.03 in MLM analysis. Ss715592224 and ss715592229 were in a region spanning 33 kb (sr5-race1), and ss715614941 and ss715614943 were in a region of 5 kb (sr13-race1). Ss715596704 located on Chr. 7 was the only SNP associated with *P*. *sojae* race 3 resistance (Table 1). It showed a significant association with LOD value of 4.40 in SMR, 4.01 in GLM, and 3.42 in MLM analysis.

Twelve SNPs on Chr. 3, 4, and 13 were associated with *P*. *sojae* race 7 resistance (Table 1). The marker, ss715588864 on Chr.4 showed the highest significant association with the resistance to race 7 with the LOD value of 5.90 in SMR, 4.72 in GLM, and 4.02 in MLM. Ss715588829, ss715588855, ss715588857, ss715588858, ss715588863, ss715588864, ss715588866, ss715588867 and ss715588868 were in a region of 58 kb (sr4-race7).

Two SNPs on Chr. 3 were associated with race 17 (Table 1) located. Ss715586846 was associated with race 17 resistance with the LOD value of 6.90 in SMR, 6.02 in GLM, and 4.18 in MLM. Ss715585114 and ss715586846 were assigned to different regions (sr3a-race17 and sr3b-race17).

Eleven SNPs on Chr. 10, 14, and 18 were associated with the race 25 (Table 1).The marker ss715619926 on Chr.14 showed the strongest association with race 25 with the LOD value of 7.97 in SMR, 7.49 in GLM, and 6.05 in MLM analysis. Seven SNPs were clustered in the 46.7 kb region. Ss715619924 and ss715619926 were in an 8.2 kb region sr10-race25. Out of the total 32 identified markers for races 1, 3, 7, 17 and 25, ss715585768 on Chr. 3 showed pleiotropic effects on both races 1 and 7 (Table 1).

Compared to previous GAWS studies on *P*. *sojae* resistance, our resistance-associated regions were either not on the same chromosomes as or had various distance from reported alleles and QTLs [9,11]. Sun et al. (2014) used 214 Chinese accessions to map partial resistance to *P*. *sojae* race 1, and identified four SSR alleles, Satt634-133, Satt634-149, Sat_222–168 and Satt301-190 on Chr. 2 and 17 [9]. The SNPs we identified for race 1 were on Chr. 3, 5, 13 and 18. Schneider et al. (2016) used 1,395 Korea accessions to identify seven QTLs on Chr. 3, 13 and 19 associated with partial resistance to *P*. *sojae* [11]. On Chr. 3, one QTL was flanked by ss715585712 and ss715585728, and spanned 49.2 kb. The ss715585768 in sr3-race1&7 region from our study was 23.9 kb from ss715585728 and 36.7 kb from ss715585712, respectively. Other resistance-associated regions were further away from QTLs identified by Schneider et al., (2016) [11].

The previous QTL mapping studies have identified 33 genomic regions on 18 chromosomes for partial resistance to *P*. *sojae* [5, 25–36]. Genome-wide association analysis of the *P*. *sojae* resistance in the Chinese soybean mini core collection showed that a total of 14 markers were significantly associated with Phytophthora resistance[10], but not all genomic regions of these SNPs were consistent with the regions reported in this study.

### Resistance-associated regions identified in the panel

The resistance-associated regions, defined as a group of significant SNPs associated with *P*. *Sojae* races, in the 189 soybean accessions were also recorded. For race 1, 78 out of the total 81 *P*. *sojae* resistant soybean accessions showed one to four resistance-associated regions. PI 377574, PI 408076C and PI 567319A, had four resistance-associated regions (S3 Table). For race 3, 42 accessions showed the resistance-associated region, sr7-race 3 (S3 Table). For race 7, 25 accessions showed one to three resistance-associated regions. Three accessions (PI 594660A, PI 417380, and PI 378682C,) had three resistance-associated regions for race 7 (S3 Table). Based on the two SNP markers of two significant regions distributed on Chr. 3 for the resistance to race 17, 10 accessions showed two favorable alleles. Based on the 11 SNP markers of three significant regions distributed on three Chrs for the resistance to race 25, 15 accessions showed resistance-associated regions, and three accessions (PI 78682C, PI 407778A, and PI 304218) had three resistance-associated regions (S3 Table). Four accessions, PI 378682C, PI 567780B, PI 567781 and PI 588021A, had resistance-associated regions for all races, showing these four PIs accessions associated with broad-spectrum resistance to *P*. *sojae*. These accessions will be highly valuable for Southern soybean breeders to be utilized for the development of *P*. *sojae* resistant varieties.

### Validation of SNP markers for *P*. *sojae* resistance

To further validate the associated SNPs, we performed gene expression analysis to characterize differentially expressed genes in response to *P*. *sojae* infection. Gene expression analyses have been widely used to identify underlying mechanisms of disease resistance for soybeans [37, 38]. To understand how many genes are differentially expressed in response to *P*. *sojae* infection and are also closely localized with the significant SNPs, we analyzed published expression data of soybean seedlings in response to *P*. *sojae* infection. For each SNP, we searched for the closest gene that was differentially expressed and we found 13 unique genes. Some genes (for example, Glyma.04g190700) were close to multiple SNPs because these significant SNPs were very close to each other due to strong LD. The distance between these significant genes and significant SNPs varied from 3 kb to 1.5 100kb. We identified three genes that were differentially expressed and were close to significant SNPs (within 100 kb distance) (Table 2. These genes included, Glyma.14g087500, which was a subtilisin protease and, was down-regulated more than 4 fold (log2 fold > 2.2) in response to *P*. *sojae* infection in our analysis. This gene was only 3,000 base pairs away from a significant SNP, ss715619920. Subtilisin proteases were well known regulators of plant-pathogen responses [39]. Another gene was Glyma.13g176600, which was a MAC/Perforin domain-containing protein. Perforin domain-containing proteins were well known in their role in immune responses [40], although little has been studied in plants. This gene was located 24 kb from a significant SNP, ss715614943, and the gene was up-regulated more than 4 fold in response to *P*. *sojae* infection. These results provided direct molecular target as candidate genes for future validation experiments.

**Table 2:** Expression analysis of differentially expressed genes from Lin et al., 2014 [24] closely-linked to significant SNPs (P<0.001) associated with *P*. *sojae* races.

SNP marker	SNPs type	Gene Name	Distance to SNP (bp)	logFC*	logCPM£	PValue¥	Lod§	FDR₤	Gene Annotationβ
ss715585768	A/G	Glyma.03g042700	1,516,790	2.81	5.17	0.00	4.21	0.01	WRKY DNA-binding protein
ss715592224	C/T	Glyma.05g238100	186,176	3.39	3.70	0.00	3.53	0.03	Diadenosine and diphosphoinositol polyphosphate phosphohydrolase
ss715592229	A/C	Glyma.05g238100	219,383	3.39	3.70	0.00	3.53	0.03	Diadenosine and diphosphoinositol polyphosphate phosphohydrolase
ss715614941	A/G	Glyma.13g176600	30,005	2.66	5.51	0.00	4.13	0.01	MAC/Perforin domain-containing protein
ss715614943	A/C	Glyma.13g176600	24,733	2.66	5.51	0.00	4.13	0.01	MAC/Perforin domain-containing protein
ss715631615	A/G	Glyma.18g261900	194,102	2.88	4.02	0.00	3.28	0.05	Predicted small molecule transporter
ss715596704	C/T	Glyma.07g023300	1,586,568	1.97	6.62	0.00	3.25	0.04	WRKY DNA-binding protein
ss715586336	A/G	Glyma.03g042700	923,940	2.81	5.17	0.00	4.21	0.01	WRKY DNA-binding protein
ss715588829	A/G	Glyma.04g190700	934,146	-2.76	5.64	0.00	4.12	0.01	Cysteine proteinases superfamily protein
ss715588855	A/G	Glyma.04g190700	1,169,785	-2.76	5.64	0.00	4.12	0.01	Cysteine proteinases superfamily protein
ss715588857	G/T	Glyma.04g190700	1,177,440	-2.76	5.64	0.00	4.12	0.01	Cysteine proteinases superfamily protein
ss715588858	G/T	Glyma.04g190700	1,186,391	-2.76	5.64	0.00	4.12	0.01	Cysteine proteinases superfamily protein
ss715588863	A/C	Glyma.04g190700	1,200,755	-2.76	5.64	0.00	4.12	0.01	Cysteine proteinases superfamily protein
ss715588864	G/T	Glyma.04g190700	1,202,470	-2.76	5.64	0.00	4.12	0.01	Cysteine proteinases superfamily protein
ss715588866	A/G	Glyma.04g190700	1,206,769	-2.76	5.64	0.00	4.12	0.01	Cysteine proteinases superfamily protein
ss715588867	G/T	Glyma.04g190700	1,223,849	-2.76	5.64	0.00	4.12	0.01	Cysteine proteinases superfamily protein
ss715588868	A/G	Glyma.04g190700	1,227,830	-2.76	5.64	0.00	4.12	0.01	Cysteine proteinases superfamily protein
ss715613726	C/T	Glyma.13g030300	714,151	6.26	2.35	0.00	3.65	0.02	lipoxygenase 2
ss715617288	C/T	Glyma.13g030300	392,083	6.26	2.35	0.00	3.65	0.02	lipoxygenase 2
ss715585114	A/G	Glyma.03g109900	223,924	2.24	8.17	0.00	4.63	0.00	Cupredoxin superfamily protein
ss715586846	C/T	Glyma.03g042700	80,281	2.81	5.17	0.00	4.21	0.01	WRKY DNA-binding protein
ss715606847	C/T	Glyma.10g161500	221,072	6.49	5.18	0.00	9.99	0.00	No annotation
ss715606853	A/G	Glyma.10g161500	213,709	6.49	5.18	0.00	9.99	0.00	No annotation
ss715606854	A/G	Glyma.10g161500	209,736	6.49	5.18	0.00	9.99	0.00	No annotation
ss715606855	C/T	Glyma.10g161500	204,489	6.49	5.18	0.00	9.99	0.00	No annotation
ss715606857	A/G	Glyma.10g161500	203,008	6.49	5.18	0.00	9.99	0.00	No annotation
ss715606859	C/T	Glyma.10g161500	192,206	6.49	5.18	0.00	9.99	0.00	No annotation
ss715606865	C/T	Glyma.10g161500	174,369	6.49	5.18	0.00	9.99	0.00	No annotation
ss715619920	A/G	Glyma.14g087500	3,014	-2.21	6.89	0.00	4.22	0.01	Subtilase family protein
ss715619924	G/T	Glyma.14g087500	17,098	-2.21	6.89	0.00	4.22	0.01	Subtilase family protein
ss715619926	A/G	Glyma.14g087500	25,344	-2.21	6.89	0.00	4.22	0.01	Subtilase family protein
ss715631508	A/G	Glyma.18g254000	227,553	5.07	3.23	0.00	3.28	0.05	Leucine-rich repeat receptor-like protein kinase family protein

*logFC: log2 fold change of gene expression.

£logCPM: log2 read count per million reads. This number provides information on gene expression level.

¥Pvalue: significant value obtained using edgeR.

§LOD: this is from GWAS analysis.

₤FDR: false discovery rate from differential expression analysis.

βGene Annotation: this showed the functional annotation of these genes. Annotations were obtained from Soybase.

## Conclusion

USDA scientists have made great effort to evaluate the U.S soybean germplasm to collect both phenotypic date (ars-grin.gov) in numerous traits and genotypic data using Illumina Infinium SoySNP50K (Soybase.org) [14]. However, we are still exploring the most efficient way to utilize the data set in order to improve the genetic base and enhance interested traits. This study used 189 accessions high in diversity for the Southern soybean production region to identify accessions with broad-spectrum resistance to *P*. *sojae* and significant regions associated with the resistance in this panel. The findings will significantly help the Southern soybean breeders to develop elite varieties with broad genetic diversity and *P*. *sojae* resistance to benefit soybean growers eventually.

## Supporting information

S1 Table Origin, agronomic information and sub-groups of the panel consisting of 189 soybean accessions. (XLSX)

S2 Table *P*. *sojae* ratings of the panel consisting of 189 soybean accessions. (XLSX)

S3 Table Resistance-associated regions for *P*. *sojae* resistance in the panel of 97 soybean accessions resistant to one or more races. (XLSX)
